# Supplementary material for: An oviduct-on-a-chip provides an enhanced in vitro environment for zygote genome reprogramming
Source: Nat Commun. 2018 Nov 22;9:4934. doi: 10.1038/s41467-018-07119-8 (PMC6250703; doi:10.1038/s41467-018-07119-8)
Supplement: Supplementary file 1 — Supplementary Information [file 41467_2018_7119_MOESM1_ESM.pdf]

Supplementary Information for "An oviduct-on-a-chip provides an enhanced *in vitro* environment for zygote genome reprogramming" by Ferraz et al.

Supplementary methods

We used two Aladdin Syringe Pumps (WPI, Germany) to deliver solutions into the microfluidic devices. Solutions were transferred into 1 ml plastic syringes (BD Biosciences, Breda, The Netherlands) and Tygon® microbore tubing (0.020" ID x 0.060" OD, Cole-Parmer, Schiedam, The Netherlands) was connected using EFD Precision tips (23 gauge, EFD Nordson, Maastricht, The Netherlands). The other end of the tubing was connected to inlets on the microfluidic device using stainless steel pins (23 gauge, New England Small Tube Co., Litchfield, NH, USA) as shown below.

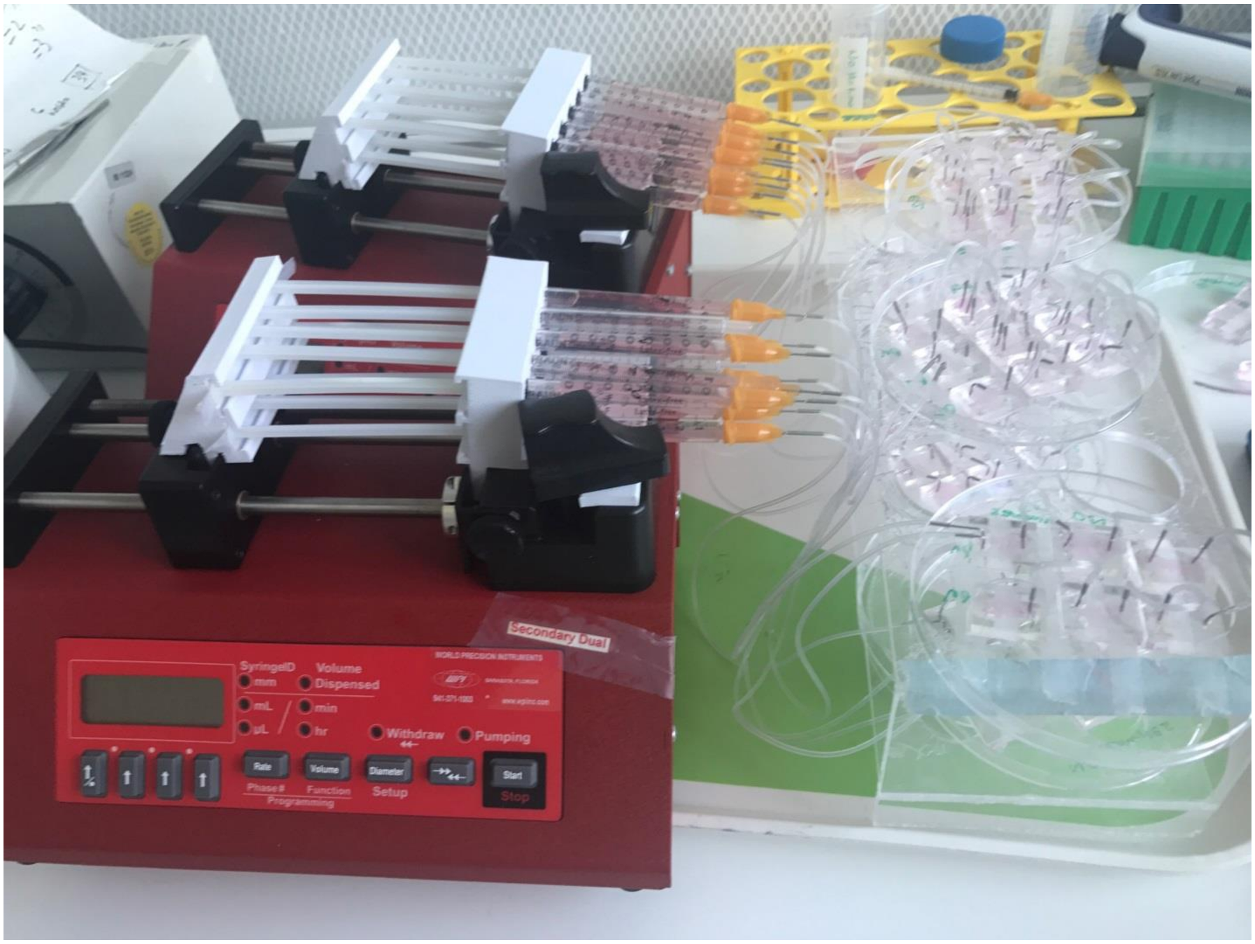

Supplementary figure 1

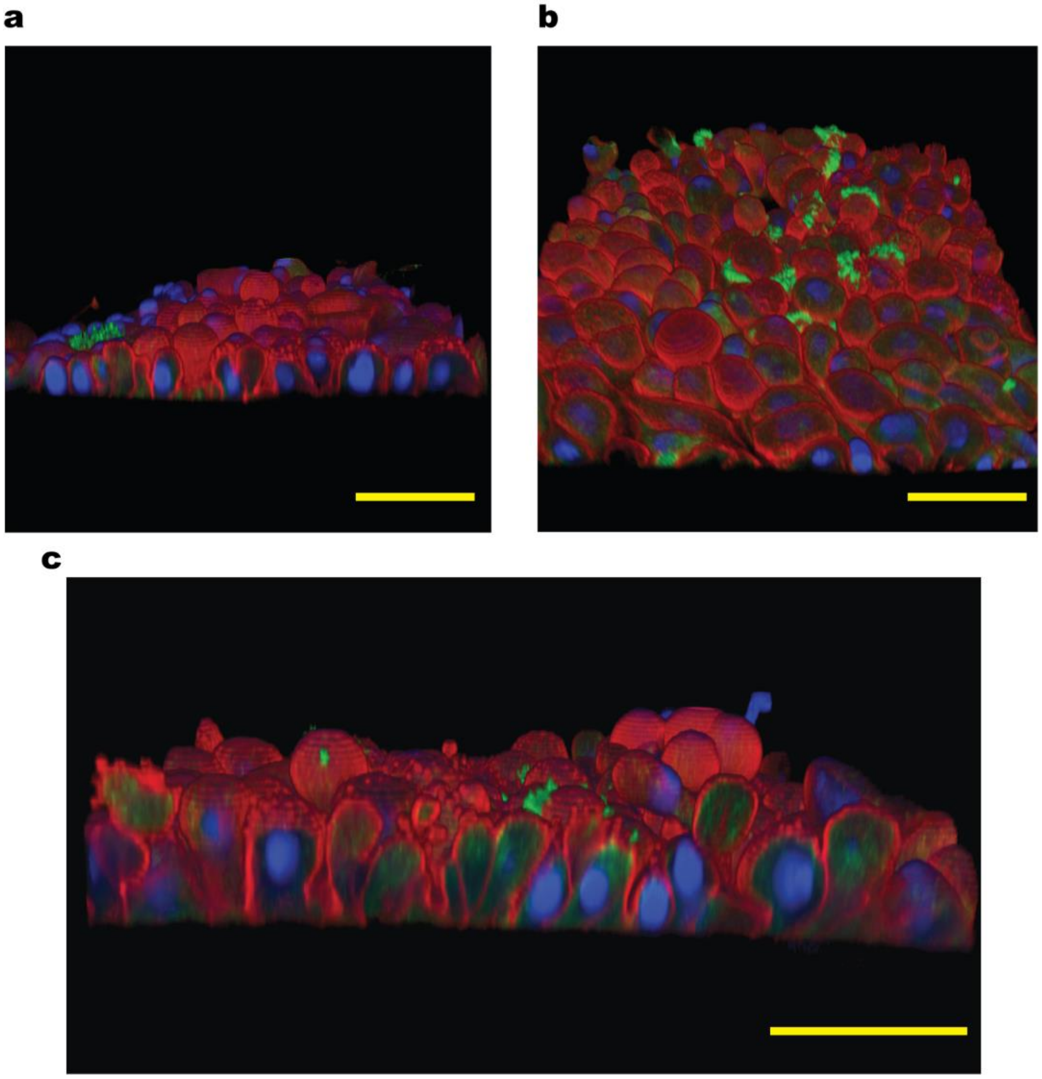

3D reconstruction of confocal immunofluorescence (IF) images for cilia (acetylated alpha-tubulin, green), nuclei (HOECHST33342, blue) and actin filaments (phalloidin, red). In (a, b and c) 3D reconstruction of part of the apical chamber, showing that BOECs lost their cuboid to columnar morphology, and started having apical protrusions, becoming a blebbing balloon shape. Scale Bars = 25 µm.

Supplementary figure 2

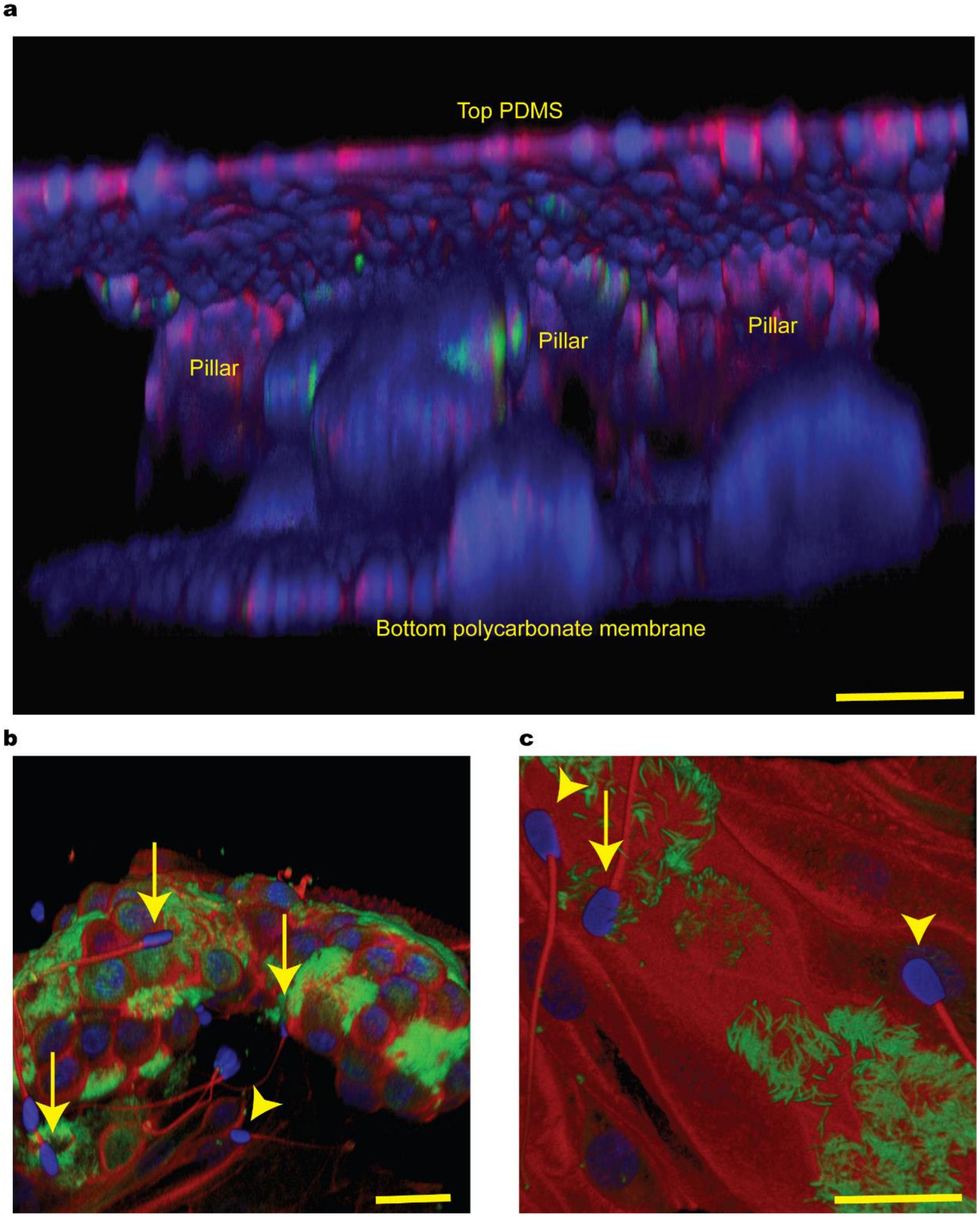

3D reconstruction of confocal immunofluorescence (IF) images for cilia (acetylated alpha-tubulin, green), nuclei (HOECHST33342, blue) and actin filaments (phalloidin, red) with and without sperm cells. (a) 3D reconstruction of part of the apical chamber, showing that BOECs grew on the overlying PDMS, on the trapping pillars and on the underlying polycarbonate membrane. Note the formation of villus-like structures, mimicking oviduct mucosal folding. (b) Closer look at a villus-like structure with attached sperm cells. Sperm cells were bound to ciliated (arrows) and non-ciliated cells (arrow heads). (c) Closer look at sperm-epithelium binding: sperm cells were bound to ciliated (arrows) and non-ciliated cells (arrow heads). Scale Bars = 50 µm (a) and 10 µm (b and c).

Supplementary figure 3

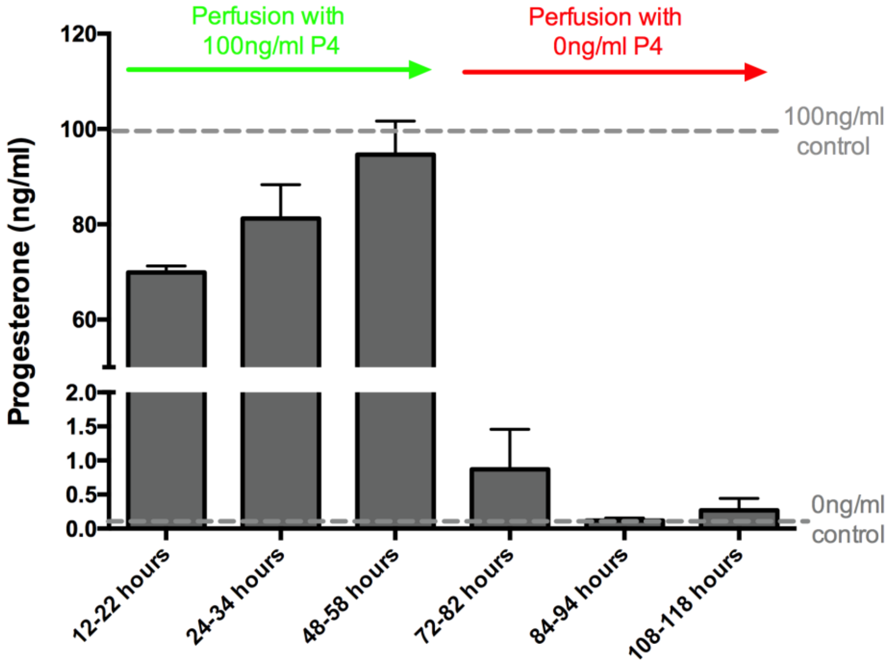

Progesterone levels (in ng ml^{-1}) measured in the fluids that passed cell free PDMS chips (n=5). The basolateral compartment was perfused at a speed of 5 uL $hour^{-1}$ with medium containing 100 ng mL^{-1} P4 (green arrow) for 60 hours. This was followed by the same medium containing no progesterone (red arrow). The fluids that came through the PDMS chips were collected in the given perfusion (total collected volume was 50 uL). The P4 levels of the collected fluids were measured for each time point in duplo (15 uL each). Mean ± SD are provided, the dashed line provides triplo measurements of the input fluids of both the 100 ng/mL and the 0 ng/mL containing media used for this perfusion experiment.

Supplementary figure 4

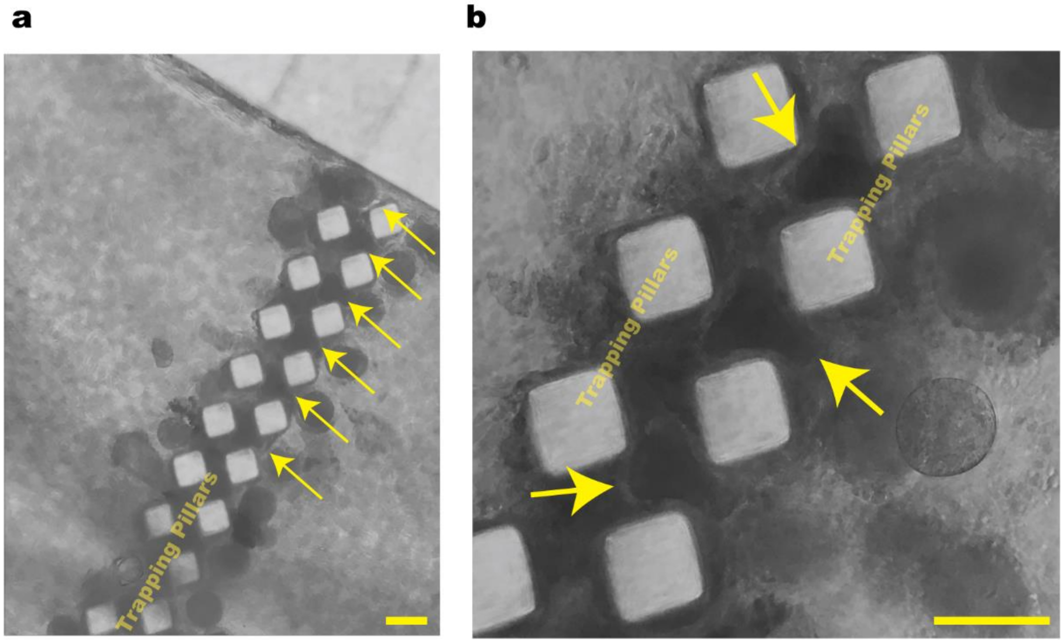

Phase-contrast images of cumulus-oocyte-complexes (COCs) inside the oviduct-on-a-chip. Note the COCs trapped (arrows) between the trapping pillars in (a) and (b). Scale bars = 100 µm.

Supplementary figure 5

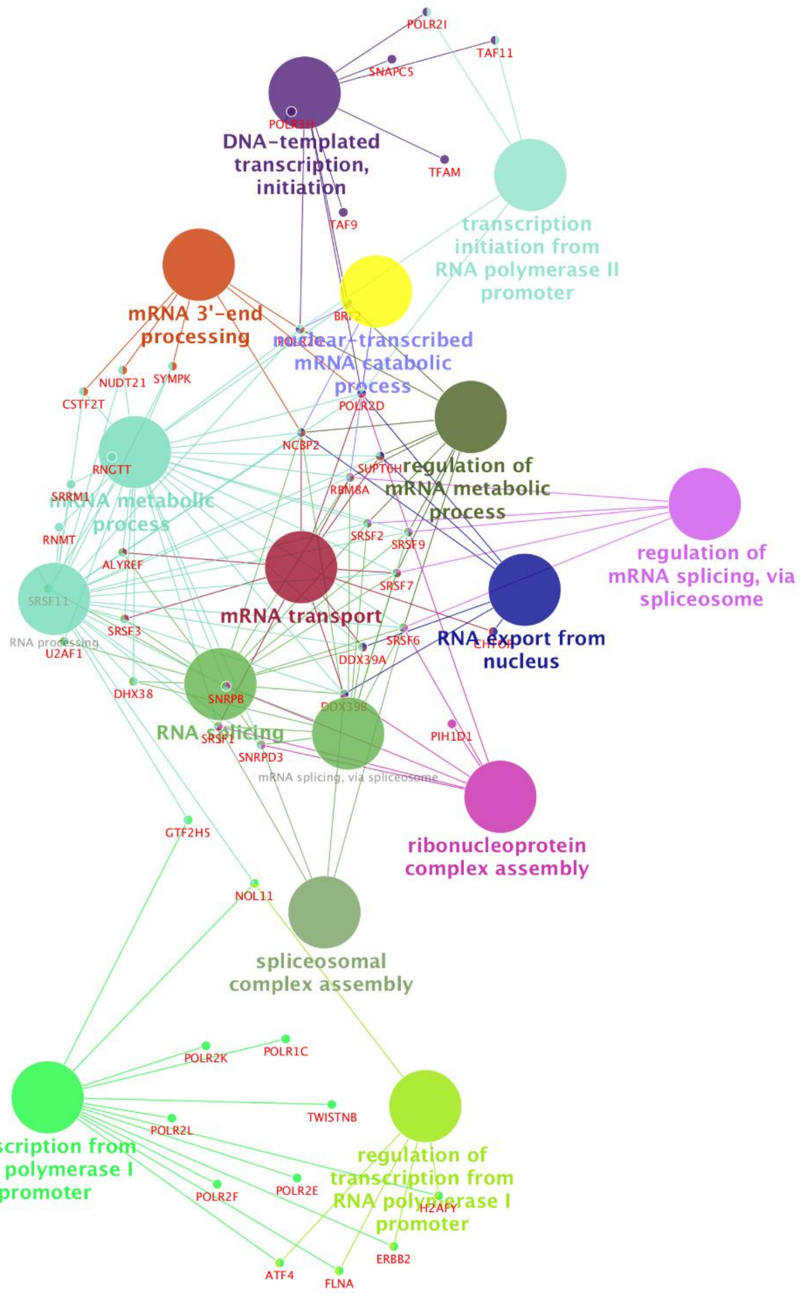

Functionally grouped gene ontology (GO) terms for genes up-regulated in G2 zygotes. The CytoScape plugin ClueGO was used to group the genes into functional GO terms of "molecular processes" and "biological processes" using genes related to transcription.

Supplementary figure 6

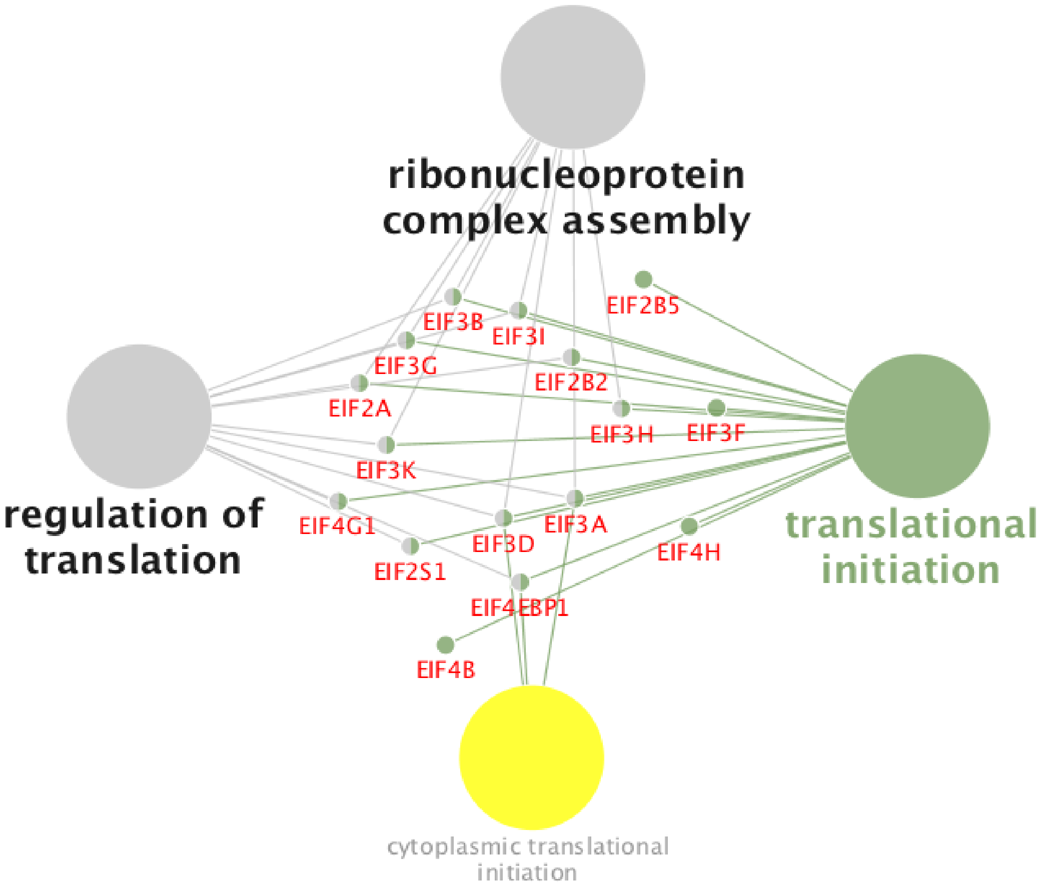

Supplementary figure 6: Functionally grouped gene ontology (GO) terms for genes up-regulated in G2 zygotes. The CytoScape plugin ClueGO was used to group the genes into functional GO terms of "molecular processes" and "biological processes" using genes related to translation initiation.

Supplementary figure 7

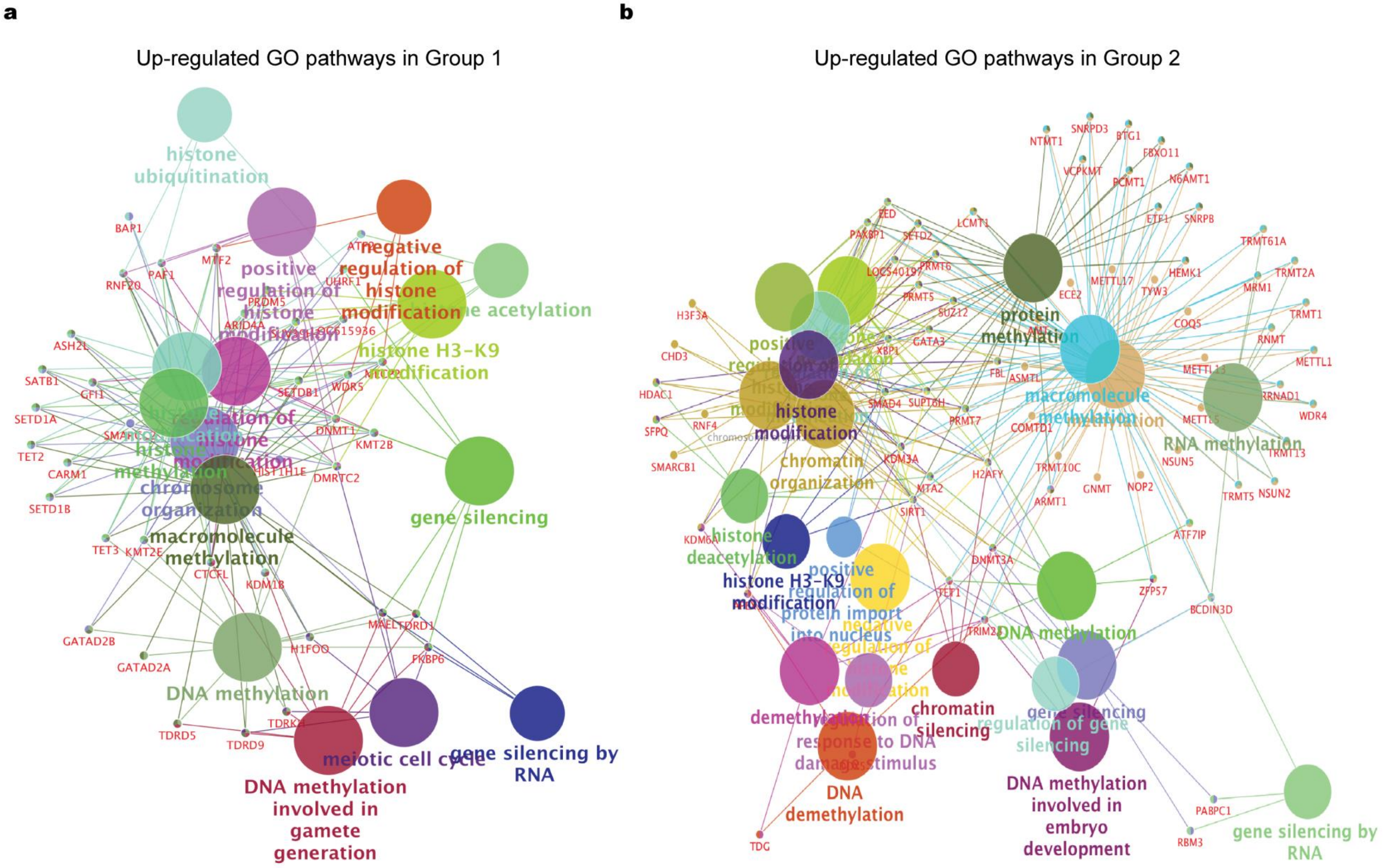

Functionally grouped gene ontology (GO) terms for genes up-regulated in G1 and G2 zygotes. The CytoScape plugin ClueGO was used to group the genes into functional GO terms of “molecular processes” and “biological processes” using genes related to (de)methylation and (de)acetylation. In (a) up-regulated GO pathways in G1 zygotes; in (b) up-regulated GO pathways in G2 zygotes.
