# Supplementary material for: An oviduct-on-a-chip provides an enhanced in vitro environment for zygote genome reprogramming
Source: Nat Commun. 2018 Nov 22;9:4934. doi: 10.1038/s41467-018-07119-8 (PMC6250703; doi:10.1038/s41467-018-07119-8)
Supplement: Supplementary file 3 — Description Of Additional Supplementary Files [file 41467_2018_7119_MOESM3_ESM.pdf]

Description of Additional Supplementary Files

File Name: Supplementary movie 1

Description: Live imaging of sperm cells incubated with BOECs cultured inside the chip. Sperm mid piece was stained with MitoTracker (red) and nuclei of both sperm and BOECs were stained by HOECHST33342 (blue).

File Name: Supplementary movie 2

Description: Reconstruction of confocal Z-stacks of the apical compartment of the chip (100x magnification). From top (PDMS wall) to bottom (polycarbonate membrane); BOECs were stained with HOECHST33342 (blue), phalloidin (red) and acetylated alpha tubulin (green). Note confluent monolayers on both top and bottom layers and also surrounding the pillars. And the presence of villus-like structures mimicking the oviductal folding.

File Name: Supplementary movie 3

Description. Imaging of COCs inside the apical compartment of the chip coincubated with sperm cells, the so called fertilization.

File Name: Supplementary data 1.

Description: Up and down regulated GO pathways

File Name: Supplementary data 2.

Description: Up regulated genes in G1 and G2
